# Detecting Cochlear Synaptopathy Through Curvature Quantification of the Auditory Brainstem Response

**DOI:** 10.3389/fncel.2022.851500

**Published:** 2022-03-09

**Authors:** Jianxin Bao, Segun Light Jegede, John W. Hawks, Bethany Dade, Qiang Guan, Samantha Middaugh, Ziyu Qiu, Anna Levina, Tsung-Heng Tsai

**Affiliations:** ^1^Department of Anatomy and Neurobiology, Northeast Ohio Medical University, Rootstown, OH, United States; ^2^Department of Research and Development, Gateway Biotechnology Inc., Rootstown, OH, United States; ^3^Department of Mathematical Sciences, Kent State University, Kent, OH, United States; ^4^Department of Computer Science, Kent State University, Kent, OH, United States

**Keywords:** cochlear synaptopathy, hidden hearing loss, noise-induced hearing loss, otitis media, Down syndrome

## Abstract

The sound-evoked electrical compound potential known as auditory brainstem response (ABR) represents the firing of a heterogenous population of auditory neurons in response to sound stimuli, and is often used for clinical diagnosis based on wave amplitude and latency. However, recent ABR applications to detect human cochlear synaptopathy have led to inconsistent results, mainly due to the high variability of ABR wave-1 amplitude. Here, rather than focusing on the amplitude of ABR wave 1, we evaluated the use of ABR wave curvature to detect cochlear synaptic loss. We first compared four curvature quantification methods using simulated ABR waves, and identified that the cubic spline method using five data points produced the most accurate quantification. We next evaluated this quantification method with ABR data from an established mouse model with cochlear synaptopathy. The data clearly demonstrated that curvature measurement is more sensitive and consistent in identifying cochlear synaptic loss in mice compared to the amplitude and latency measurements. We further tested this curvature method in a different mouse model presenting with otitis media. The change in curvature profile due to middle ear infection in otitis media is different from the profile of mice with cochlear synaptopathy. Thus, our study suggests that curvature quantification can be used to address the current ABR variability issue, and may lead to additional applications in the clinic diagnosis of hearing disorders.

## Highlights

- We developed a new method to detect cochlear synaptopathy using curvature quantification.
- We identified that the cubic spline method applied to five data points produces the most accurate curvature quantification of ABR waves.
- We demonstrated better detection ability using this curvature method than the currently accepted amplitude method.

## Introduction

In neurodegenerative diseases, synaptic loss often occurs before obvious functional changes. Loss of synapses in Alzheimer’s disease starts years before symptoms appear, which contributes to mild cognitive impairment (John and Reddy, 2021). A similar early synaptic loss occurs in both the peripheral and central auditory systems (for recent reviews, Bharadwaj et al., 2019; Ibrahim and Llano, 2019; Kujawa and Liberman, 2019). If synaptic loss could be diagnosed by non-invasive detection tools, risk factors favoring degenerative mechanisms could be identified in earlier stages, and the loss of the ability to manage daily living activities could be delayed (for reviews, Mukherjea et al., 2011; Henstridge et al., 2016; Spankovich and Yerraguntla, 2019). The seminal discovery of cochlear synaptopathy was initially made in CBA/CaJ mice following moderate noise exposure (Kujawa and Liberman, 2009). They showed, using a precise quantification of synaptic loss by means of immunocytochemistry, the loss of up to half of the synapses between inner hair cells (IHCs) and spiral ganglion neurons (SGNs), despite full recovery of hearing thresholds as measured by auditory brainstem response (ABR). Subsequent detailed analyses identified noise conditions that led to a significant permanent decrease in the supra-threshold growth of ABR wave I amplitude, despite a full recovery of distortion product otoacoustic emission (DPOAE) amplitudes (Furman et al., 2013; Fernandez et al., 2020). The DPOAE measurement is important because it eliminates possible confounding effects on the ABR wave I from damage to outer hair cells (OHCs).

Currently, there are no validated non-invasive clinical tools or battery of test to detect cochlear synaptopathy in humans (Bramhall et al., 2019). Pure-tone audiometry is still the primary tool for detecting hearing loss. However, clinicians often find that patients within normal audiometric thresholds complain of difficulty hearing in noise and other auditory perceptual anomalies (Plack et al., 2016). Hidden hearing loss (HHL) is used to describe this condition (Schaette and McAlpine, 2011). Although behavioral tests may be possible to detect cochlear deafferentation (e.g., Lobarinas et al., 2020), an ABR based detection method could be ideal for detecting auditory dysfunction because it is already used in clinical settings and a decreased ABR wave-I amplitude is associated with cochlear synaptopathy in animal studies (Kobel et al., 2017). In humans there are five prominent ABR waves that are labeled wave I, II, III, IV, and V, with the wave I and II have been correlated with auditory nerve function of the distal and more proximal portions, respectively (for review, Hall and Rupp, 1997). Five similar waves can be observed in animal models (Waves 1–5). Based on previous animal studies, HHL could be due to cochlear synaptopathy of SGNs with predominantly low spontaneous rate (SR) and high thresholds, which likely leads to no detectable changes in audiometric thresholds (Furman et al., 2013). Recent data from CBA/CaJ mice further confirmed this noise-induced synaptic loss, but demonstrated a synaptic loss of both low- and high-SR SGNs (Suthakar and Liberman, 2021). In spite of this emerging evidence of cochlear synaptopathy in rodents, primates (Valero et al., 2017) and post-mortem human tissues (Wu et al., 2019), functional determination of this synaptic loss in humans is not conclusive (e.g., Fulbright et al., 2017; Grinn et al., 2017; Prendergast et al., 2019). By comparing participant groups exposed to either low or high amounts of noise, a correlation between ABR or electrocochleography (ECochG) wave I amplitude and estimated noise exposure has been found in some studies but not in others (Stamper and Johnson, 2015a; Liberman et al., 2016; Bramhall et al., 2017; Guest et al., 2018). Most importantly, even if there is a consistent correlation from the population data, for clinical use, we still need a sensitive detection method for individual diagnosis. Failure of consistent detection with these methods is mainly due to high variability in wave-I amplitude. The high variability in human ABR/ECochG wave I in population studies is due to a number of factors, such as head size, sex, and genetic heterogeneity (Stamper and Johnson, 2015b; Bharadwaj et al., 2019). This variability can be reduced by using longitudinal studies of individual subjects, similar to preclinical studies (Kujawa and Liberman, 2009). However, for individual diagnosis, random electrical noise such as brain and muscle electrical activities can still lead to high variability in ABR wave-I amplitude across repeated measurements. In addition, the ABR wave-I amplitude is not sensitive to the loss of low-SR SGN fibers, due to the delayed and broad first-spike latency distribution of low-SR fibers (Bourien et al., 2014).

Besides wave amplitude, the shape of ABR/ECochG waves may provide additional information regarding synaptic loss of SGNs. Wave I comes from the summed response of a mixed population of SGN fibers. Due to the effects of the averaging process used with ABR/ECochG, the shape of the wave I averaged response is dependent on the conduction velocity and fiber diameter of the contributing neurons, which vary depending on each fiber’s type and location in the cochlea (e.g., Liberman, 1982). High-SR and low-threshold SGN fibers have shorter first-spike latencies than SGN fibers with low-SR and high-threshold fibers (Heil and Irvine, 1997). Compared with high-SR and low-threshold SGNs, low-SR and high-threshold fibers have larger dynamic firing ranges, longer first-spike latencies, and slower conduction velocities (Liberman, 1978; Heil and Irvine, 1997). Subsequently, different SGN fibers may contribute to different parts of ABR wave I shape. Thus, one possible solution to the high variability issue of wave I amplitude is to quantify the shape characteristics of the wave using curvature instead of their amplitudes. Here, we first compared four curvature quantification methods using simulated ABR waves, and identified that the cubic spline method using five data points produced the most accurate quantification. We next evaluated this quantification method with ABR data from an established mouse model of cochlear synaptopathy. Our curvature measurement can quantify curvature changes of three areas of ABR wave 1: the right curve, the peak, and the left curve. This method is much more sensitive and consistent in identifying cochlear synaptic loss in mice than the amplitude and latency measurements. We further tested this curvature method in a different mouse model of conductive hearing loss, and found a different curvature profile for early hearing loss due to middle ear infection in mice with otitis media. This suggests that this curvature method is sensitive to detect cochlear deafferentation, and it is promising to detect other types of hearing loss based on its different curvature profiles.

## Materials and Methods

### Ethics Statement

All mouse studies were approved by the Institutional Animal Care and Use Committee of Northeast Ohio Medical University (NEOMED) in accordance with the National Institutes of Health guidelines.

### Animals

The animal data were collected from 4-month-old CBA/CaJ mice (*n* = 58, 31 males) and 8–9-week-old Ts65Dn mice (*n* = 12, 6 males) obtained from the Jackson Laboratory (Bar Harbor, ME, United States). For CBA/CaJ mice, after initial ABR and DPOAE threshold testing was conducted to ensure normal hearing, mice were randomly assigned to one of two groups: a control group without noise exposure, and a 96-dB group exposed to a band of noise (8–16 kHz) at 96 dB SPL for 2 h. Mice were then held for 2 weeks post exposure before repeating ABR and DPOAE threshold testing, as well as ABR click testing. For the Ts65Dn mice, similar ABR threshold, ABR click, and DPOAE threshold testing were conducted, as well as tympanometric measurements to assess for otitis media.

### Noise Exposures

A free-field noise exposure was used whereby each mouse was unrestrained in a sub-divided cage within a foam-lined, double-walled, sound-isolated room (Industrial Acoustics, North Aurora, IL, United States). The band of noise (8–16 kHz) was generated with custom LabVIEW software, and routed through a power amplifier (Crown CDi1000) to a loudspeaker (Selenium D3500Ti-Nd, JBL, Northridge, CA, United States). Before each exposure, noise levels were calibrated to 96 dB SPL, and during the exposure, the noise level was continually monitored at the center of the cage using a B&K 4153 1/4-inch microphone connected to an amplifier (1–100,000 Hz; Bruel & Kjaer Nexus Amplifier).

### Auditory Physiologic Tests

Similar to our previous studies (e.g., Bao et al., 2004, 2013), mice were anesthetized with a solution of ketamine and xylazine (80/15 mg/kg, i.p.) and positioned dorsally in a custom head holder. Mouse body temperature was maintained at 37.5 ± 1.0°C using an isothermal pad. DPOAEs were measured using an ER10B+ microphone/pre-amplifier (Etymotic Research) and processed with a TDT RZ6/BioSigRZ system (Tucker-Davis Technologies). DPOAEs were elicited with two pure tones, f1 and f2, using an f2/f1 ratio of 1.2, where F2 = 20 kHz, with emissions collected for levels from 90 to 0 dB SPL in 5 dB steps. One hundred sweeps were presented at each test frequency. Input/output functions of DPOAE were quantified and the threshold of DPOAE was defined as the level at which a response could be noted at least 5 dB SPL above the noise floor.

For all ABR testing, three electrodes were placed subdermally behind the test ear (active), the vertex (reference), and the base of the tail (ground). Evoked potentials were collected using a Tucker Davis Technology (TDT) RZ6 processor and BioSigRZ software. Thresholds were obtained by presenting tone bursts at 5, 10, 20, and 40 kHz from 90 dB SPL descending in 5 dB steps to 0 dB SPL or 10 dB below threshold. Tones were 5 ms in duration, 0.5 ms rise/fall, with a repetition rate of 17.1/s, with potentials averaged over 512 repetitions. Threshold was defined as the level where any ABR wave could be identified. ABR responses were also obtained using click stimuli with an initial onset of 0.1 ms presented at 70 and 90 dB peSPL. Clicks were 0.1 ms in duration, of alternating polarity with a repetition rate of 1.9/s. Evoked potentials were averaged over 1024 repetitions and band-pass filtered (100–3,000 Hz).

### Curvature Quantification

**FIGURE 1:**
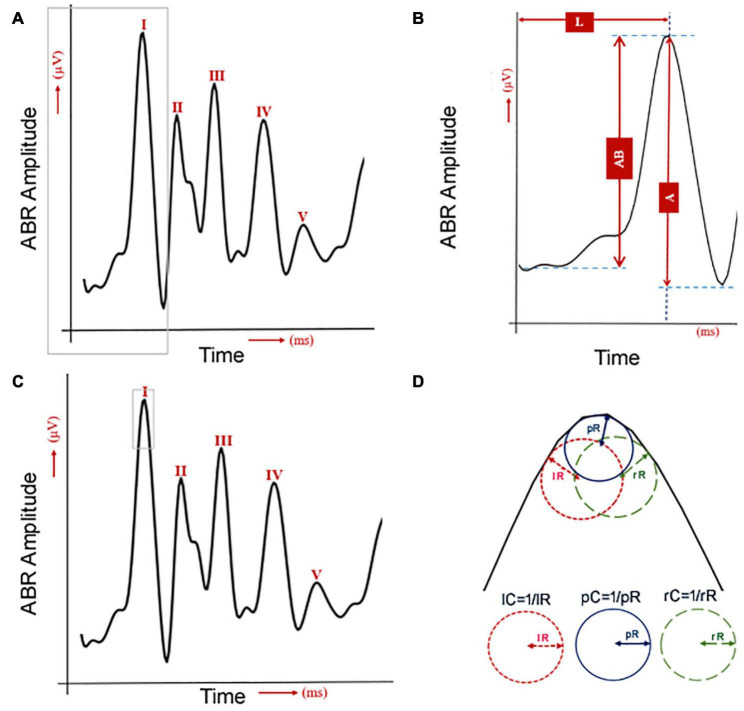
Quantification of six ABR wave-I features. **(A)** One human ABR sample with five waves. The gray lines were used to label the wave I, which is used for the next panel. **(B)** The human ABR wave 1 was commonly analyzed by measurements of latency (L) and amplitude. The amplitude was quantified either by the peak to the baseline (AB) or the peak to the trough (A). **(C)** The same human ABR sample with five waves with the gray lines to label the area for curvature analysis in panel **(D)**. **(D)** Three curvature quantifications for the shape of ABR wave I.

For the data collected from the click sound stimulation, a computational workflow implemented in Python was used to process the ABR data (Figure 1): peak amplitude from trough (A), peak amplitude from baseline (AB), peak latency (L), peak curvature (pC), left curvature (lC), and right curvature (rC). For curvature calculations, four methods were considered (Stoer and Bulirsch, 1992): non-linear least squares (NLLS), Lagrange polynomials (LGP), numerical differentiation (ND), and cubic spline (CS). For a particular data point from an ABR wave, (*x, y*), the curvature of this point is defined as


$$k=\frac{\left|x^{\prime }\,y^{\prime \prime }-y^{\prime }\,x^{\prime \prime }\right|}{{\left[{\left(x^{\prime }\right)}^{2}+{\left(y^{\prime }\right)}^{2}\right]}^{3/2}}$$

where the numerical differentiation method finds the partial derivatives based on *n* data points around (*x, y*). When *y* is considered as a function of *x*, *y* = *f*(*x*), the above formula is simplified to


$$k=\frac{\left|f^{\prime \prime }\,\left(x\right)\right|}{{\left[1+{\left(f^{\prime }\,\left(x\right)\right)}^{2}\right]}^{3/2}}$$

### Statistical Analysis

Following feature quantification, statistical analyses were performed using R^1^ to determine the significance of the difference in curvature, amplitude and latency changes between pre- and post-noise exposures (for CBA/CaJ mice) and that between normal and mice with otitis media (for Ts65Dn mice). For detecting possible changes of ABR wave-1, we quantified a total of six features (Figure 1). ABR wave-I amplitude was measured in two ways: from the baseline (AB) or from the next trough (A). Its latency was measured from the sound stimulation onset to the wave-I peak (L) (Figures 1A,B). Its peak (pC), left (lC), and right curvature (rC) were also quantified (Figure 1D). Since two ABR data collections (pre- and post-noise exposure) were made for each CBA/CaJ mouse, a paired *t*-test was applied to evaluate the mean difference in quantified features between the pre- and post-noise exposure. In the detection of hearing loss in otitis media mice, two separate groups of mice (control vs. otitis media) were analyzed and a two-sample *t*-test was used. For all the differential analyses, a significance level of 0.05 was considered.

## Results

### Comparison Among Four Curvature Quantification Methods

**FIGURE 2:**
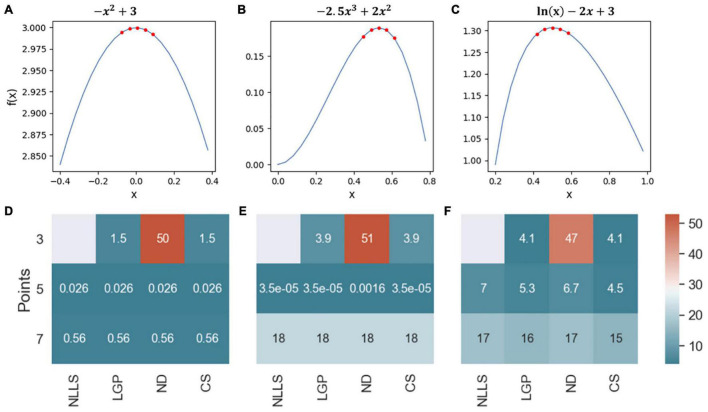
Comparison of four curvature measurements with simulated ABR waves. **(A–C)** Three simulated ABR waves. The five red dots at the peak illustrated how the peak curvature was quantified with five data points. **(D–F)** Three heatmaps were used to summarize the quantification accuracy among four quantification methods.

To identify the best curvature quantification method for ABR waves, we first compared the four curvature quantification methods with three simulated ABR waves (Figures 2A–C). The three simulated curves were considered for the following characteristics observed in ABR waves: (1) symmetric and non-symmetric shapes, and (2) sampling rate of 24,414.0625 Hz to mimic our ABR recording system (Tucker-Davis Technologies, United States). To cover all possible wave changes around wave peaks, curvature quantification was performed for three, five, or seven points. The quantification accuracy was based on the percent absolute error of the calculated curvature value and the results are summarized in a heatmap (Figures 2D–F). For the first simulated ABR curve, the accuracy for all four measurements was the same except for the ND method with 3 data points (Figure 2D). For the other two simulated waves, the CS method with five data points produced either equal to or the most accurate results than the other curvature quantification methods. Based on these simulation results, we subsequently chose the CS approach.

### Development of Mouse Model for Noise-Induced Cochlear Synaptopathy

Based on previous detailed studies of cochlear synaptopathy in the same mouse strain (e.g., Fernandez et al., 2020; Suthakar and Liberman, 2021), we assigned CBA/CaJ mice to one of two groups: control or noise-exposed (96 dB SPL for 2 h). Hearing function of the mice was quantified by both ABR and DPOAE methods before and after the noise exposure to detect temporary threshold shift (TTS; 1 day after noise exposure) and permanent threshold shift (PTS; 2 weeks after noise exposure). Three-factor ANOVAs, with frequency and time as within-subjects effects, revealed a group difference for both ABR threshold [*F*(12, 432) = 30.7130; *p* = 2.0787E-43] and DPOAE threshold [*F*(12, 417) = 33.6826; *p* = 8.4233E-46]. For control mice (*n* = 19; 10 males) without noise exposure, no significant TTS or PTS were observed. In contrast, for noise-exposed mice (*n* = 25, 13 males), post-hoc analyses revealed TTS at 10 kHz (*p* = 0.0009, ABR threshold; *p* = 0.0058, DPOAE threshold), 20 kHz (*p* = 7.271E-21, ABR threshold; *p* = 8.8521E-32, DPOAE threshold), and 40 kHz (*p* = 7.2229E-52, ABR threshold; *p* = 1.7373E-52, DPOAE threshold). The 2-week thresholds of the noise-exposed mice were not significantly different from the pre-exposure thresholds, indicating that none sustained PTS. Thus, mice from the 96-dB noise group had TTS, but no PTS.

**FIGURE 3:**
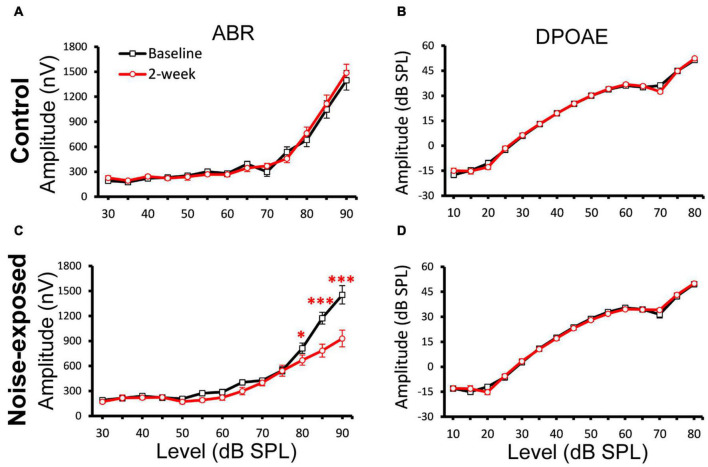
A decrease of ABR Wave-1 supra-threshold amplitudes only in the noise-exposed group. ABR and DPOAE at 20 kHz were tested for both the control and noise-exposed mice before (Baseline) and 2-week after the noise exposure. **(A)** No changes of ABR amplitudes between the baseline and post 2-week measurement in the control mice. **(B)** No change of DPOAE amplitudes in the control mice. **(C)** A significant decrease of ABR amplitudes between the baseline and post 2-week measurement for the noise-exposed mice. **(D)** No change of DPOAE amplitudes in the noise-exposed mice. * *p* < 0.05. ^***^ *p* < 0.01. Data shown are the means ± 1 SEM.

In mouse models, it is established that cochlear synaptopathy is highly correlated with reduced ABR wave-1 amplitudes at supra-threshold levels once OHC functions have recovered following noise exposure (Furman et al., 2013; Fernandez et al., 2015, 2020). We subsequently quantified ABR wave-1 and DPOAE amplitude in the same two mouse groups (Figure 3). A significant decrease in ABR wave-1 amplitude with suprathreshold sound stimulation was not found in the control group (Figure 3A), but was found in the noise-exposed group 2 weeks post noise exposure (Figure 3C). Three-factor ANOVAs (group x time x level), with time and stimulus level as within-subjects effects, indicated a significant reduction of ABR wave-1 amplitude at 20 kHz 2 weeks post exposure [*F*(26, 1034) = 3.9049; *p* = 1.1221E-9]. Bonferroni’s multiple comparisons test at each sound intensity/time combination revealed a significant difference at 80 dB SPL (*p* = 0.0233), 85 dB SPL (*p* = 1.196E-9) and 90 dB SPL (*p* = 7.4444E-16), while no significant changes in DPOAE amplitude were found for either the control (Figure 3B) or the noise-exposed group (Figure 3D). Thus, the noise-exposed group showed a typical phenotype of cochlear synaptopathy.

### Comparison of Curvature and Amplitude Quantification

**FIGURE 4:**
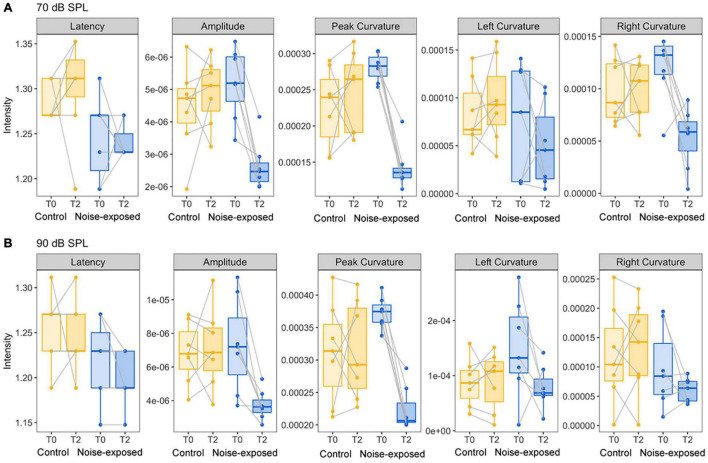
Comparison of five ABR features between control mice and noise-exposed mice. **(A)** For the ABR wave 1 evoked by 70 dB SPL, a decreased of ABR amplitude, peak and right curvature quantifications were found only for mice post noise exposure at 96 dB SPL. **(B)** For the ABR wave 1 evoked by 90 dB SPL, decreases of ABR amplitude, peak, and left curvature quantifications were found only for mice post noise exposure at 96 dB SPL.

**FIGURE 5:**
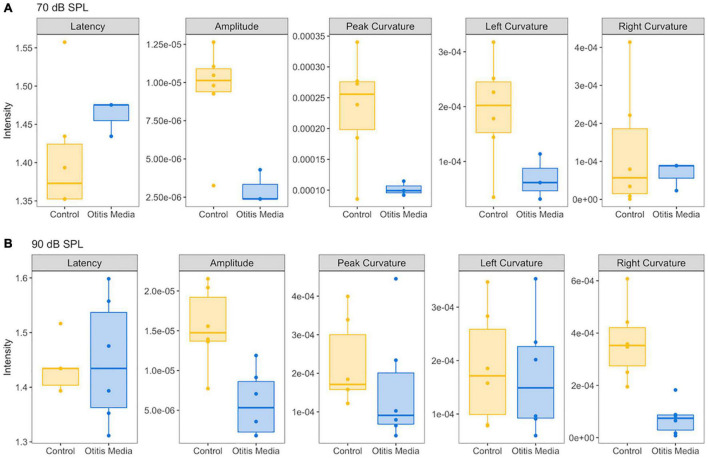
Comparison of five ABR features between control mice and mice with otitis media. **(A)** For the ABR wave 1 evoked by 70 dB SPL, compared to control mice, a decrease of ABR amplitude, peak and left curvature were found for mice with otitis media. **(B)** For the ABR wave 1 evoked by 90 dB SPL, decreases of ABR amplitude and right curvature were found for mice with otitis media.

To determine which features of ABR wave-1 are strongly associated with cochlear synaptopathy, we compared six quantitative features from ABR data collected with 70 or 90 dB SPL sound stimulation from the same mice pre-noise exposure and 2 weeks post-noise exposure (Figure 4). No obvious changes in latency were observed for either group of mice (Figures 5A,B first panel). Both amplitude measurements (A and AB) showed similar results, thus, we only present results for the peak-to-trough amplitude (A). At 70 dB SPL (Figure 4A), for the control group, no obvious changes in these six features were observed between the baseline and 2-week post exposure data, as expected. In contrast, for the noise-exposed group, the amplitude measurement (A) showed a decreased amplitude with a small amount of overlap between pre- and post-exposure data points (the second panel). In contrast, a decreased peak curvature was observed for every noise-exposed mouse with no overlap between pre- and post-noise exposure data (the third pane). A trend of decreased curvature post-exposure was also observed for both the left (lC; the fourth panel) and right sides (rC; the fifth panel) of ABR wave-1. At 90 dB SPL (Figure 4B), no differences in these features were observed for the control group. In contrast, for the noise-exposed group, a decreased ABR amplitude (A) was observed with an overlap between pre- and post-exposure, while the pC for the same wave was clearly decreased for every noise-exposed mouse post-exposure. A similar but less consistent trend of curvature decrease was also found for both lC and right rC measures. The same data were statistically analyzed. With 70 dB SPL stimulation (Table 1), for the noise-exposed group, the difference in amplitude (A) was significant between pre- and post-noise exposure (*p* = 0.0008), while the pC quantification provided a better separation between pre- and post-noise exposure (*p* < 0.0001). We also found a significant difference for the noise-exposed group in right curvature (rC) between pre- and post-noise exposure (*p* = 0.0153). With 90 dB SPL stimulation (Table 2), for the noise-exposed group, a significant difference in amplitude (A) was noted between pre- and post-noise exposure (*p* = 0.0096), while an even more significant difference in the pC measurements between pre- and post-noise exposure was observed (*p* = 0.0002). In short, at both sound intensities, the curvature measurements were more sensitive than the amplitude measurements in detecting cochlear synaptic loss.

**TABLE 1:** Result of the paired *t*-test for the difference between pre- and post-noise exposure at the 70 dB SPL.

Group	Feature	*t*-statistic	df	*p*-value
Control	L	–0.4714	6	0.6540
Control	A	–0.8989	6	0.4033
Control	pC	–0.6170	6	0.5599
Control	lC	–0.7160	6	0.5009
Control	rC	–0.0772	6	0.9409
Noise-exposed	L	0.2810	6	0.7882
Noise-exposed	A	6.2403	6	0.0008*
Noise-exposed	pC	9.5886	6	< 0.0001*
Noise-exposed	lC	0.8323	6	0.4371
Noise-exposed	rC	3.3555	6	0.0153*

L = latency, A = amplitude, pC = peak curvature, lC = left curvature, rC = right curvature, and df = degrees of freedom. The t-statistic is the ratio of the mean feature difference (T0–T2) to its standard error. A p-value less than the significance level (0.05) is labeled with a *.

**TABLE 2:** Result of the paired *t*-test for the difference between pre- and post-noise exposure at the 90 dB SPL.

Group	Feature	*t*-statistic	df	*p*-value
Control	L	0.2810	6	0.7882
Control	A	–0.3453	6	0.7417
Control	pC	–0.1089	6	0.9168
Control	lC	–0.1201	6	0.9083
Control	rC	–0.4275	6	0.6839
Noise-exposed	L	1.4412	6	0.1996
Noise-exposed	A	3.7451	6	0.0096*
Noise-exposed	pC	8.2330	6	0.0002*
Noise-exposed	lC	1.9179	6	0.1036
Noise-exposed	rC	1.3439	6	0.2276
				

L = latency, A = amplitude, pC = peak curvature, lC = left curvature, rC = right curvature, and df = degrees of freedom. The t-statistic is the ratio of the mean feature difference (T0–T2) to its standard error. A p-value less than the significance level (0.05) is labeled with a *.

### Curvature Quantification in Detecting Hearing Loss of Otitis Media

In order to determine if these observed curvature profiles were specific to cochlear synaptopathy, we repeated the same method in a mouse model of otitis media. Since about 70% of mice with Down syndrome from the Ts65Dn transgenic mouse model develop otitis media at 3 months old (Han et al., 2009), we performed similar ABR and DPOAE threshold tests on Ts65Dn mice at 8–9 weeks old. Among 12 mice tested, six had normal ABR and DPOAE thresholds and tympanograms within the range of normal, and the remainder presented with acute otitis media (AOM). DPOAE thresholds for all AOM mice were below the noise floor and five of the six had elevated ABR thresholds. A statistically significant difference was only found for ABR thresholds (*p* = 0.004) between the control and AOM mice (Table 3). Interestingly, quantitative analysis of their ABR wave-1 click responses showed a pattern different from normal for mice with acute otitis media (Figure 5). With 70 dB SPL stimulation (Table 4), there were significant differences between the control and AOM mice in amplitude (A; *p* = 0.0037), pC (*p* = 0.0143), and lC (*p* = 0.0332), while with 90 dB SPL stimulation, significant differences were only observed for amplitude (A; *p* = 0.0052) and rC (*p* = 0.0032), Thus, the curvature profile for otitis media, which is mainly due to middle ear dysfunction, was different from the curvature profile for cochlear synaptopathy.

**TABLE 3:** Tympanometry, DPOAE and ABR data of Ts65Dn transgenic mice.

Group	SC (ml)	TP (-daPa)	DPOAE (dB)	ABR (dB)
Control	2.32	9	40	25
Control	1.32	35	45	30
Control	1.32	43	45	25
Control	1.13	11	45	40
Control	1.15	47	45	25
Control	1.68	19	40	25
Otitis Media	1.16	70	N/A	50
Otitis Media	0.8	27	N/A	55
Otitis Media	0.74	12	N/A	55
Otitis Media	0.83	37	N/A	35
Otitis Media	1.56	20	N/A	65
Otitis Media	0.91	23	N/A	35
*p*-value	0.06	0.71	N/A	0.004*

Static compliance (SC) and tympanometry pressure (TP) were obtained from tympanometry. Both DPOAE and ABR data were collected at 20 kHz, a sensitive hearing region for mice. For each measurement, a two-sample t-test between control mice and mice with otitis media was performed. A p-value less than the significance level (0.05) is labeled with a *.

**TABLE 4:** Result of the two-sample *t*-test for the difference in feature measurements between control and otitis media groups.

Levels	Feature	*t*-statistic	df	*p*-value
70 dB	L	–1.53	6.40	0.173
70 dB	A	4.36	6.69	0.0037*
70 dB	pC	3.58	5.33	0.0143*
70 dB	lC	2.65	6.99	0.0332*
70 dB	rC	0.854	5.97	0.426
90 dB	L	–0.271	6.49	0.795
90 dB	A	3.59	9.65	0.0052*
90 dB	pC	0.842	9.17	0.421
90 dB	lC	0.249	9.99	0.809
90 dB	rC	4.50	6.78	0.0030*

L = latency, A = amplitude, pC = peak curvature, lC = left curvature, rC = right curvature, and df = degrees of freedom. A p-value less than the significance level (0.05) is labeled with a *.

## Discussion

To address the current failure of detecting cochlear synaptopathy by ABR/ECochG methods, we developed a new method to quantify possible changes in ABR/ECochG wave-1. Instead of relying on amplitude measurements, we focused on curvature measurements, and identified that the cubic spline method calculated with five data points as the most accurate method for assessing changes of ABR wave 1. Using a well-established mouse model of cochlear synaptopathy, we demonstrated that these curvature measurements are more sensitive and consistent in identifying individual mice with cochlear synaptic loss compared to amplitude measurements.

Our findings have directly addressed the high variability of ABR/ECochG wave-I amplitude, which is a major current obstacle in applying ABR/ECochG methods for human diagnosis. Although curvature quantification is well established in other fields, no studies of its application are reported for ABR/ECochG data analysis. Potential advantages of our new approach are: (1) less influenced by background noise compared to more traditional wave peak amplitude analyses, and (2) potentially more sensitive to cochlear synaptic loss of low-SR SGN fibers. Both of these advantages may be the reasons underlying our finding that the curvature method is much more sensitive than the amplitude method. In addition, the change of curvature profiles are different between the mouse model with cochlear synaptopathy and the mouse model with otitis media. Early hearing loss of otitis media is due to the middle ear infection without a significant damage to the cochlea (Trune and Zheng, 2009). Its significant curvature changes are that pC and lC to sound clicks at 70 dB SPL, and only rC to sound clicks at 90 dB SPL. In the mouse model with noise-induced cochlear synaptopathy, pC values are reduced to sound clicks to both 70 and 90 dB SPL, and the rC value is reduced only at 70 dB SPL. Since the major difference between these two animal models is a different cause of hearing loss: synaptic damages vs. middle ear infection, different curvature profiles of ABR wave 1 is most likely associated with these two causes, respectively. However, it would be better to validate by other models of hearing loss. For example, we have found that early hearing loss in cisplatin-induced hearing is associated with mitochondrial loss in SGNs only (Chen et al., 2021), and it would be interesting to apply the same method to identify curvature profiles in this model. In addition, most of noise-induced hearing loss in humans is less likely to cause purely syanptopathy, and may include other cochlear damages such as loss of OHCs (e.g., Fernandez et al., 2020); noise exposure can also create early damage to the extreme base of the cochlea which may influence mass-stiffness properties of the basilar membrane. All these damages may cause possible changes of ABR wave-1 shape. Additional studies are needed to validate our method in these models.

There are several limitations of our study. Due to central neural plasticity (Gold and Bajo, 2014; Lewis et al., 2015), we are aware that cochlear synaptopathy could lead to central plasticity changes, which would result in curvature changes of other ABR waves. In the future, it is worth to carry out a multi-metric approach to the curvature quantification of all major ABR/ECochG waves that reflect activity in both peripheral and central auditory areas (McClaskey et al., 2020). An intrinsic technical issue with this approach would be the reliable identification of multiple functional features associated with cochlear synaptopathy *via* subjective analyses of ABR/ECochG waveform tracings. Recently, machine learning has been developed as an effective statistical technique for identifying multiple features associated with complex phenomena and has been successfully applied in auditory research (e.g., Bramhall et al., 2018). Thus, machine learning may be tested to identify key features in ABR/ECochG waveforms. The other major weakness is a lack of human validation study. It is our general strategy to improve ABR/ECochG data collection and analysis first in well-established animal models, and then validate them in future human studies.

## Data Availability Statement

The original contributions presented in the study are included in the article/supplementary material, further inquiries can be directed to the corresponding author.

## Ethics Statement

The animal study was reviewed and approved by the Institutional Animal Care and Use Committee of NEOMED.

## Author Contributions

JB, SJ, JH, QG, and T-HT were responsible for the experimental design. JB, SJ, JH, BD, SM, ZQ, AL, and T-HT were responsible for data analysis. JB, JH, and T-HT were responsible for providing overall ideas. All authors contributed to the article and approved the submitted version.

## Conflict of Interest

JB, JH, BD, and ZQ were employed by Gateway Biotechnology Inc., for this project. The remaining authors declare that the research was conducted in the absence of any commercial or financial relationships that could be construed as a potential conflict of interest.

## Publisher’s Note

All claims expressed in this article are solely those of the authors and do not necessarily represent those of their affiliated organizations, or those of the publisher, the editors and the reviewers. Any product that may be evaluated in this article, or claim that may be made by its manufacturer, is not guaranteed or endorsed by the publisher.

## Footnotes

1
https://www.R-project.org
